# The Neuroprotection of 1,2,4‐Triazole Derivative by Inhibiting Inflammation and Protecting BBB Integrity in Acute Ischemic Stroke

**DOI:** 10.1111/cns.70113

**Published:** 2024-11-05

**Authors:** Xuan Liu, Jingning Luo, Jianwen Chen, Ping Huang, Gongyun He, Xueshi Ye, Ruiqi Su, Yaoqiang Lao, Yang Wang, Xiangjun He, Jingxia Zhang

**Affiliations:** ^1^ Department of Medicinal Chemistry, School of Pharmaceutical Science Sun Yat‐Sen University Guangzhou China

**Keywords:** 1,3,5‐triphenyl‐1,2,4‐triazole derivative, blood–brain barrier, glycocalyx, inflammatory

## Abstract

**Background:**

The oxidative stress and neuroinflammation are important factors in acute ischemic stroke (AIS). Our former study showed the 1,2,4‐ triazole derivative (SYS18) had obviously neuroprotection by anti‐ oxidative stress on rat middle cerebral artery occlusion (MCAO) model.

**Aim:**

In this study, we continue to investigate its neuroprotection by anti‐inflammatory effects and protecting BBB integrity in AIS.

**Methods and Results:**

First, its effect on acute inflammation was evaluated by the mice model of increased peritoneal capillary permeability. Then, the MCAO cerebral edema models were built to evaluate its neuroprotection by reducing the neurological score, cerebral edema, improving the biochemical indicators, and pathological damage of brain tissue. At the same time, its protection on blood–brain barrier (BBB) integrity was proved by decreasing the BBB permeability and inhibiting glycocalyx degradation and regulating the BBB tight junction proteins expression of matrix metalloproteinase‐ 9 (MMP‐ 9) and claudin‐ 5 in brain tissue. Meanwhile, pharmacokinetic experiments showed that the compound had good BBB penetration. It has some advantages in the intensity of efficacy compared with the marketed drug edaravone.

**Conclusion:**

Based on these findings, SYS18 has a strong potential to become a neuroprotectant in the future.

## Graphical abstract

SYS18 has a certain therapeutic effect on acetic acid–induced acute inflammation. It protects the BBB integrity by decreasing the BBB permeability, inhibiting endothelial glycocalyx degradation, and regulating TJs and MMP‐9 expression levels in AIS. SYS18 can alleviate neurological impairment by inhibiting inflammation and protecting the BBB integrity in AIS.

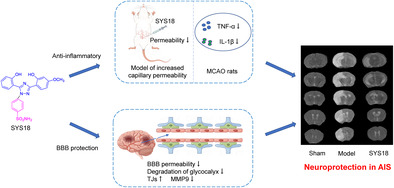

**Abbreviations**
AIS: acute ischemic stroke
B/P: ratio of total drug concentration in brain to plasma
BBB: blood–brain barrier
COX: cyclooxygenase
EDA: edaravone
EG: endothelial glycocalyx
HIF‐1: hypoxia inducible factor‐1
HPSE: heparinase
HS: heparan sulfate
IF: immunofluorescence
IL‐1β: interleukin 1 beta
iNOS: inducible nitric oxide synthase
MCAO: middle cerebral artery occlusion
MDA: malondialdehyde
MMP‐9: matrix metalloproteinase‐9
MRI: magnetic resonance imaging
PCs: pericyte cells
ROS: reactive oxygen species
SOD: superoxide dismutase
TJs: tight junctions
TNF‐α: tumor necrosis factor alfa

## 1 Introduction

Acute ischemic stroke (AIS) is a serious neurological disease in clinic. The oxidative stress and neuroinflammation are important factors in AIS [1]. The oxidative stress generates excessive free radicals which damage brain tissues and the BBB [2], and damaged BBB results the infiltration of peripheral inflammatory and immune cells to enter the ischemic brain regions and exacerbate neuroinflammation and cerebral edema in AIS [3]. Therefore, inhibition of oxidative stress and neuroinflammation maybe a potential strategy of drug research of AIS in clinic [4].

Blood–brain barrier permeability is affected by BBB integrity; in addition, endothelial glycocalyx (EG) has been shown to regulate BBB permeability [5, 6]. EG is polysaccharide‐protein composition in the vascular lumen that is abundantly expressed on endothelial cells of the BBB. The severity of stroke is related to glycocalyx dysfunction. Glycocalyx shedding can lead to the damage of vascular endothelial molecules, resulting in BBB destruction, brain edema formation, and neurological deterioration in rats [7, 8, 9].

Blood–brain barrier is closely linked with inflammation and oxidative stress. The BBB is located in the brain parenchyma and blood circulation, and controls the entry of blood substances into the brain through paracellular and transcellular pathways [10]. The BBB is composed of tightly packed single‐layer cerebral capillaries, including cell types such as microvascular endothelial cells, pericytes, astrocytes, microglia, and neurons [11]. Among them, microvascular endothelial cells mainly connect with other cells through tight junctions (such as claudin‐5) and adhesive junctions, regulating paracellular pathways; pericytes are mainly responsible for the transcytosis pathway [12].

When inflammation occurs, immune cells in the brain secrete pro‐inflammatory cytokines, and the structure of the BBB tends to loosen, including the disruption of pericyte cells (PCs) that regulate phagocytosis and tight junctions (TJs) that regulate paracellular pathways. Cells in the brain secrete TNF‐α, which then activates the NF‐κB pathway to secrete MMP‐9, disrupting the tight junction protein and basement membrane of the blood–brain barrier [13]; TNF‐α outside the brain can disrupt the BBB through two pathways: activating the cyclooxygenase (COX) pathway to increase COX‐2 secretion, which in turn leads to an increase in MMP‐9 [13]; increasing the release of inducible nitric oxide synthase (iNOS) can damage microvascular endothelial cells and increase BBB permeability. The elevated level of another inflammatory factor interleukin 1 beta (IL‐1β) stimulates astrocytes to secrete hypoxia inducible factor‐1 (HIF‐1), leading to BBB degradation, while also promoting the release of other inflammatory factors such as TNF‐α and IL‐6 [14].

When there is an imbalance between the pro oxidative molecules in the body and the antioxidant capacity of cells, oxidative stress occurs, most typically resulting in the production of excessive reactive oxygen species (ROS). ROS can attack cellular macromolecules such as proteins, lipids, and nucleic acids, leading to cellular damage. At this point, other active oxidative substances inside the cell increase, which can activate the inflammatory response, causing infiltration of inflammatory cells and release of inflammatory mediators [15].

**FIGURE 1:**
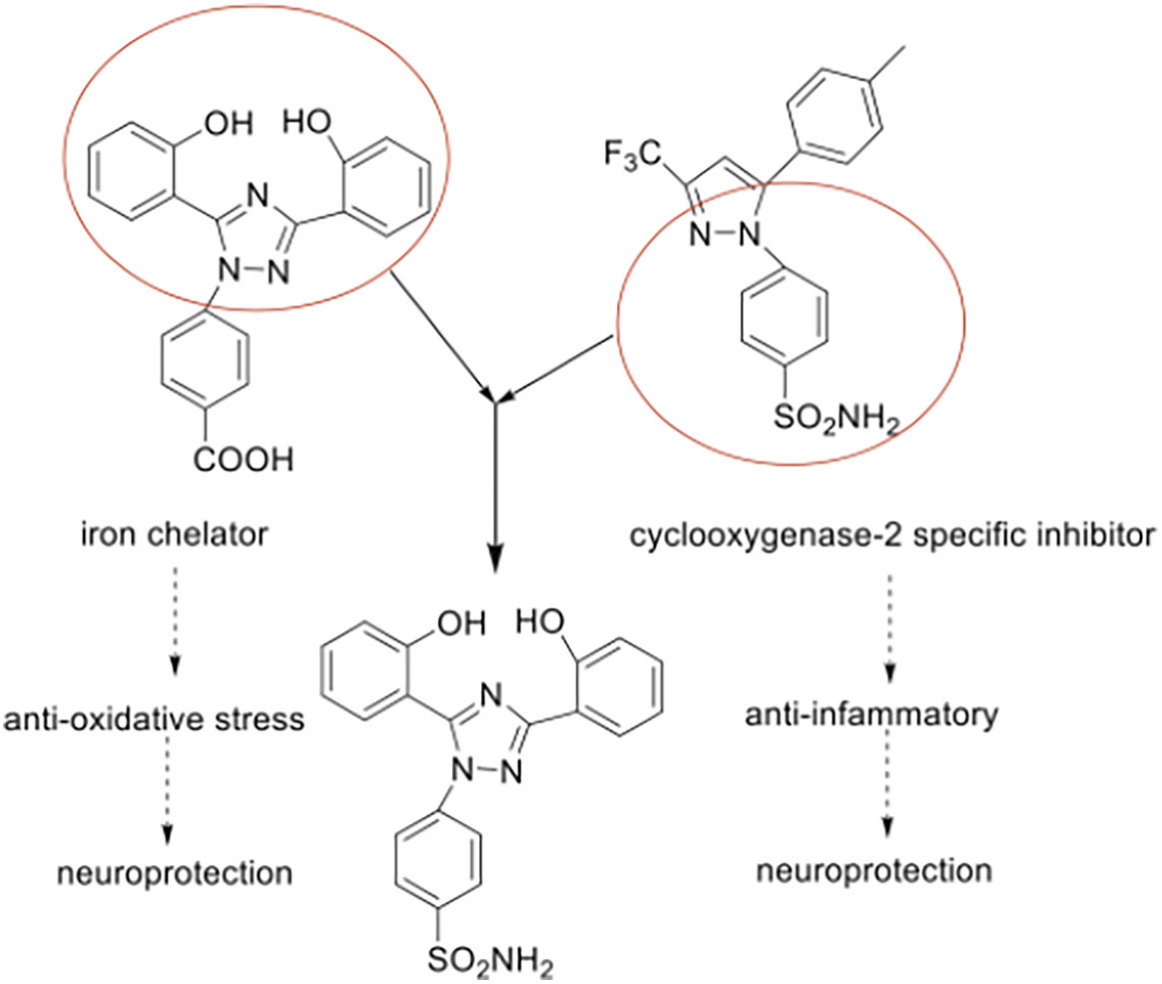
Design of triazole derivatives.

Deferasirox contains bisphenol group and triazole core as effective fragments, which have effective anti‐oxidative stress actions [16]; celecoxib has anti‐inflammatory actions, and its benzenesulfonamide and pyrazole ring are the effective fragments [17]. We had used the combination principles to design and synthesize a series of 1,3,5‐triphenyl‐1,2,4‐triazole derivatives and proved the compound SYS18 is the optimal one with effective anti‐oxidative stress action (in the original article, SYS18 is referred to as Compound **11**) (Figure 1) [18].

The former research results showed SYS18 has neuroprotective effects on nitroprusside (SNP)‐induced PC12 injury and acute ischemic stroke model by anti‐oxidative stress [18]. In this study, we continue to validate the neuroprotective effect of SYS18 by anti‐inflammatory, especially its role in reducing brain edema and histological damage of nerve cells. We next evaluated the protective effects of SYS18 on glycocalyx and BBB. Here, the cerebral‐blood distribution of SYS18 and the pharmacokinetic parameters in the brain are also described.

## 2 Materials and Methods

### 2.1 Materials and Reagents

#### 2.1.1 Instruments

Embedding machine (JB‐P5, Wuhan Junjie Electronics Co. Ltd.), pathological slicer (RM2016, Shanghai Leica Instruments Co. Ltd.), chemiluminescence imaging system (Tanon5200, Shanghai Tianneng Co. Ltd.), and cell imaging system (EVOS FL Auto, Life Technologies Co. Ltd., USA).

#### 2.1.2 Reagents

MCAO Wiring (A4‐26365‐250‐280 g, Beijing Xinong Technology Co. Ltd.), rat tumor necrosis factor‐α (TNF‐a) ELISA kit (RX302058R, China Ruixin Biotech Co. Ltd.), rat interleukin‐1β (IL‐1β) ELISA kit (RX302869R, China Ruixin Biotech Co. Ltd.), superoxide dismutase (SOD) kit‐WST‐8 method (RXWB0477‐96, China Ruixin Biological Technology Co. Ltd.), MDA detection kit (RXWB0005‐96, China Ruixin Biological Technology Co. Ltd.), isopropanol, chloroform (Shanghai McLean Biochemical Technology Co. Ltd., China), DEPC water (Beijing G‐CIONE Biotechnology Co. Ltd.), MMP‐9 rabbit monoclonal antibody (ab76003, abcam, UK), claudin‐5 rabbit polyclonal antibody (YT09523, Immunoway, US), sheep anti‐rabbit IgG/HRP(ab181602, US, abcam, UK), rat heparan sulfate ELISA kit (RX303120R, China Ruoxin Biotech Co. Ltd.), rat Heparanase (HPSE) ELISA kit (RX39254, China Ruoxin Biotech Co. Ltd.), rat syndecan‐1 ELISA kit (RXSY196, China Ruixin Biotechnology Co. Ltd.), TUNEL kit (11684817910, Roche Life Science Co. Ltd., USA), and DAB chromogener (K5007, DAKO, Germany).

#### 2.1.3 Main Reagent Preparation Methods

(1) Preparation of 10 mg/mL SYS18 solution: 20 mL of polyoxyethylene castor oil was diluted into 20% concentration by adding 80 mL of normal saline. In total, 0.5000 g SYS18 was dissolved in 50 mL of 20% polyoxyethylene castor oil by ultrasound. (2) Preparation of Evans Blue: 2.0000 g Evans blue was dissolved in 50 mL normal saline to prepare 4% Evans blue solution.

#### 2.1.4 Animals

Kunming mice, C57BL/6 mice, and Sprague–Dawley rats weighing 200–230 g (6–7 weeks old) were purchased from Sun Yat‐sen University Laboratory Animal Center. The animals were kept in quarantine for at least 3 days before the experiment. Animals were housed in a room at 21°C–22°C, 45%–50% humidity, and 12‐h light. They had free access to food and water. All experimental procedures were performed in strict accordance with the guidelines of the Ethics Committee of Sun Yat‐sen University (Guangzhou, China) in experimental procedure. The protocol was approved annually by the Animal Experimentation Committee of Sun Yat‐sen University (approval number: SYSU‐IACUC‐2021‐000613).

### 2.2 The Synthesis of 4‐(3‐(2‐Hydroxy‐4‐Methoxyphenyl)‐5‐(2‐Hydroxyphenyl)‐1H‐1,2,4‐Triazol‐1‐yl) Benzenesulfonamide (SYS18)

4‐Methoxysalicylic acid (10 mmol, 1.0 equivalent), salicylamide (11 mmol, 1.1 equivalent), and a catalytic amount of pyridine were added to 15 mL anhydrous xylene and heated until dissolved. Thionyl chloride (20 mmol, 2.0 equivalent) was added carefully to the solution with stirring. Then, the reaction was carried out at 140°C for 3 h and monitored by TLC. After cooling to room temperature, the crude product was collected by filtration. After stirring in ethanol at 75°C for 2 h, the intermediate was collected by filtration and dried under vacuum. The intermediate (1 mmol, 1 equivalent) and hydrazine derivative (1.2 mmol, 1.2 equivalent) were added to 5 mL of ethanol. The reaction was carried out at 75°C for 6 h and monitored by TLC. After completion of the reaction, the crude product was adsorbed on silica, followed by purification via flash chromatography (1.7 g, yield: 78.0%). m. p. 254°C–256°C. ^1^H NMR (500 MHz, DMSO‐d_6_) d 10.73 (s, 1H), 9.59 (s, 1H), 8.05 (d, *J* = 7.7 Hz, 1H), 7.89 (d, *J* = 8.1 Hz, 2H), 7.65 (d, *J* = 8.0 Hz, 2H), 7.50 (s, 2H), 7.38 (t, *J* = 7.9 Hz, 1H), 7.13 (s, 1H), 7.05–6.97 (m, 3H), 6.79 (dd, J = 8.9, 2.4 Hz, 1H), 3.73 (s, 2H). ^13^C NMR (126 MHz, DMSO‐d_6_) d 160.4, 156.8, 152.5, 152.4, 149.4, 144.2, 140.7, 132.0, 127.3, 127.2, 124.3, 120.2, 119.2, 117.6, 117.6, 115.7, 114.9, 114.1, 56.0. HRMS (m/z): calculated for C_21_H_19_O_5_N_4_S [M + H]^+^ 439.1071, found 439.1074.

### 2.3 Establishment of a Mice Model of Increased Peritoneal Capillary Permeability

#### 2.3.1 Experimental Grouping

Sixty Kunming mice were randomly divided into control group, SYS18 low, medium, and high‐dose groups (20, 40, and 80 mg/kg, respectively), and positive control group was given celecoxib (40 mg/kg), 10 mice in each group. The mice in each group were administrated with 0.2 mL/10 g once a day for three consecutive days.

#### 2.3.2 Model of Increased Peritoneal Capillary Permeability Induced by Acetic Acid

One hour after the last administration, 2% Evans blue saline 0.1 mL/10 g was injected via the tail vein, and 0.6% acetic acid 0.2 mL/10 g was injected intraperitoneally immediately. Twenty minutes later, the mice were killed, the abdominal cavity was cut open, and the abdominal cavity was washed several times with 5 mL normal saline to collect washing fluid. The samples were centrifuged at 4700 r/min for 5 min, and the lateral absorbance at the wavelength of 620 nm was measured with a UV spectrophotometer to compare the differences between groups.

### 2.4 Establishment of a Mice Model of Ischemic Stroke and Measurement of Brain Edema Volume

#### 2.4.1 Procedures for Middle Cerebral Artery Occlusion

C57BL/6 mice were randomly assigned to treatment groups by body weight. Operator blind to treatment status of animals. After the mice were anesthetized, a midline cervical incision was made. The left common carotid artery and external carotid artery were isolated and ligated. Arterial clamps were placed at the preparation line and distal end of the internal carotid artery. An incision was made at the bifurcation of the common carotid artery. 4–0 nylon line was inserted to a depth of 17–20 mm. The nylon line entered the internal carotid artery; then, it entered the skull to the anterior cerebral artery and blocked all blood flow sources in the middle cerebral artery. After keeping ischemic for 1 h, the animals were re‐anesthetized and then pulled out the nylon line for about 1 cm to achieve reperfusion for up to 48 h. The wound was disinfected with iodophor, and the skin was sutured [19]. Put them on the electric blanket to maintain the body temperature after the completion of surgery; then, they were returned to cages upon recovery. For the sham group, except that no nylon line is inserted, other steps are the same as above. Sham group and model group were given 20% polyoxyethylene castor oil. Subsequently, the animals were sacrificed, and the serum and brains were collected for a series of subsequent analyses and studies.

#### 2.4.2 Measurement of Brain Water Content

Serial brain images were acquired approximately 48 h after MCAO. C57BL/6 mice were divided into sham operation group, MCAO model group, and treatment intervention group, with five mice in each group. SYS18 was administered at a dose of 30 mg/kg twice a day. All experimental procedures were performed as described above (Procedures for middle cerebral artery occlusion). T2 Image mode, five consecutive slices of 1.0 mm thickness scanning, FOV 25 × 25 mm, TR value 3000 ms, TE value 69.12 ms, and Image J software analysis of brain edema volume percentage.

### 2.5 Establishment of a Rat Model of Ischemic Stroke and Measurement of Brain Water Content

#### 2.5.1 Experimental Grouping

Rats were randomly divided into five groups: Sham group (*n* = 24), rats received sham operation; MCAO model group (*n* = 30); SYS18 low‐dose group (7.5 mg/kg, *n* = 30); SYS18 high‐dose group (15 mg/kg, *n* = 30); and Positive control group rats were intraperitoneally injected with edaravone (15 mg/kg, *n* = 30). The tested compound was dissolved in 20% polyoxyethylene castor oil and was administered 0.5 h before operation and 6, 12, 24, and 36 h after reperfusion. The measurements were carried out 48 h after the operation. Ten animals in each group were used to measure brain water content, and 10 animals in each group were used to detect Evans blue content in brain tissue. Ten animals in each group were used for determination of biochemical indicators and pathological observations and WB experiments.

#### 2.5.2 Procedures for Middle Cerebral Artery Occlusion

All experimental procedures were performed as described above (2.4. Establishment of a mice model of ischemic stroke).

#### 2.5.3 Measurement of Brain Water Content

The brain water content was evaluated by measuring difference between the dry and wet weights of brains. Briefly, after rats were sacrificed after 48 h of reperfusion, the brains were measured immediately to obtain the wet weights. Then, the brains were drying in a constant temperature oven at 100°C for 24 h, followed by measurements to obtain the dry weights. Brain water content was calculated according to the following formula, brain water content = 100% × (wet weight − dry weight)/wet weight [20].

### 2.6 Evaluation of Neurological Score

According to Zea‐Longa 4‐point scoring criteria, the animals were scored for neurological function at 48 h after surgery. As a successful model, the operated side exhibited significant Horner's disease and hemiplegic somatic disease (the left forelimb could not be fully extended and left while walking). And the degree of each animal model was scored as follows: 0 point, no symptoms of nerve injury; 1 point, inability to fully extend the forepaw; 2 points, circling laterally; 3 points, tip to the opposite side; 4 points, inability to walk spontaneously and loss of consciousness [21]. The selection criteria for this article are a neurobehavioral score of 1 or above, with symptoms of hemiplegia or anterior limb weakness.

### 2.7 Hematoxylin and Eosin Staining

Hematoxylin and eosin staining were used to observe the effect of SYS18 on pathological changes in ischemia–reperfusion brain tissue. After 48 h of ischemia–reperfusion, the rats were sacrificed, and the brains were immediately fixed with 4% paraformaldehyde after transcranial perfusion with ice‐cold saline (The damaged brain is located in the ischemic penumbra of the right brain. Through HE staining and pathological sectioning under a microscope, the area where neuronal cells are morphologically intact and have not yet degenerated or necrotic is determined as the ischemic penumbra). Paraffin sections were deparaffinized to water, and stained with hematoxylin and eosin, dehydrated and mounted. Sections were observed and photographed in an Eclipse Ti‐S inverted microscope at 400× [22].

### 2.8 Determination of Biochemical Indicators

Blood sample was collected at 48 h after MCAO. After centrifugation at 4°C × 12,000 g for 10 min, the clear supernatant was stored at −80°C. The components of SOD, MDA, TNF‐α, and IL‐1β in the blood samples were measured according to the instructions of the assay kits.

### 2.9 Assessment of Blood–Brain Barrier Permeability

The integrity of the BBB was investigated using Evans blue (EB). Forty‐six hours after reperfusion, rats were injected with EB (4% saline, 4 mL/kg) via the tail vein. Two hours later, rats were anesthetized and perfused with saline through the left ventricle until colorless perfusate. After hydrangea and brainstem were removed, the brains were collected and homogenized. Evans blue was extracted from the brain with *N*,*N*‐dimethylformamide. After centrifugation at 3700 rpm for 15 min, the absorbance of supernatant was detected at 620 nm wavelength [23].

### 2.10 Measurement of Glycocalyx Components

Blood and brain sample were collected at 48 h after MCAO. After centrifugation at 4°C × 12,000 g for 10 min, the clear supernatant was stored at −80°C. Their concentrations of syndecan‐1, HS, and heparanase (HPSE) were determined using an ELISA kit. In brief, add the calibrator and the sample to be tested into a microplate pre‐coated with solid phase antibody, and then add another HRP‐labeled corresponding antibody (enzyme‐labeled antibody). After incubation and sufficient washing, remove the unbound components, and form a solid phase antibody antigen–enzyme‐labeled antibody sandwich complex on the solid phase surface of the microplate. Substrates A and B were added, and the substrate was catalyzed by HRP to produce a product, which eventually converted under the action of a stop solution, and the absorbance (OD value) was measured at a wavelength of 450 nm on a microplate reader.

### 2.11 Immunofluorescence Staining

Immunofluorescence testing selects the same area as HE staining to observe and analyze the fluorescence level in the field of view. After deparaffinization of the paraffin sections to water, the tissue sections were subjected to antigen retrieval in a microwave oven in a retrieval box filled with citric acid antigen retrieval buffer (pH 6.0), and the sections were incubated overnight at 4°C with rabbit monoclonal anti‐MMP‐9 (1:100), rabbit polyclonal anti‐claudin‐5 (1:200). Incubate for 50 min in the dark with CYS3 goat anti‐rabbit antibody (1:500), DAPI staining solution was added dropwise and incubated for 10 min at room temperature in the dark. Finally, an inverted scanning microscope was used to observe at 400×.

### 2.12 Blood–Brain Distribution and PK Determination of SYS18 in Rats

In 21 SD rats, SYS18 was injected intraperitoneally at 15 mg/kg. The rats were fasted for 15 h before the experiment, forbidden to drink water for 2 h after administration, fasted for 4 h, and were fed a uniform meal during the experiment. At 0, 1, 2, 4, 6, 12, and 24 h after administration, three rats were selected for blood collection from the posterior orbital venous plexus and whole brain extraction. The blood samples were anticoagulated with EDTA and stored at 4°C, then centrifuged at 4°C at 4000 r/min for 10 min. The plasma concentrations were detected simultaneously (plasma stored at −80°C refrigerator). The whole brain was stored at −80°C for later detection and then uniformly prepared into brain tissue homogenate to detect drug concentration. Use Excel to analyze the drug concentration—time data pharmacokinetic parameters through atrioventricular method.

### 2.13 Statistical Analysis

The experimental data were statistically processed by GraphPad Prism 8.0 biostatistics software. Results were expressed as mean ± SD. The *Q*‐value test was used to exclude suspicious data. Measurement data satisfying normality were analyzed by two independent samples *t*‐test or one‐way ANOVA combined with Dunnett's multiple comparison method. The sham group and the model group were analyzed by two independent samples *t*‐test, and model group and each experimental group were analyzed, respectively, by one‐way ANOVA. Measurement data that did not satisfy normality and enumeration data were analyzed by Mann–Whitney *U* test or Kruskal–Wallis rank sum test. *p* Value less than 0.05 was considered statistically significant.

## 3 Results

### 3.1 The Chemical Structure of SYS18

**FIGURE 2:**
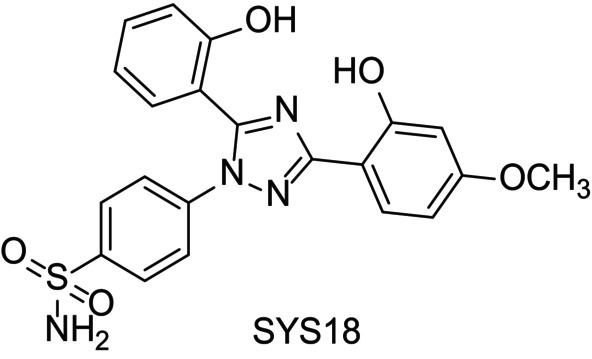
The chemical structure of SYS18.

The chemical structure of SYS18 is 4‐(3‐(2‐hydroxy‐4‐methoxyphenyl)‐5‐(2‐hydroxyphenyl)‐1H‐1,2,4‐triazol‐1‐yl) benzenesulfonamide (abbreviation, SYS18, Figure 2). The target product was purified by column chromatography, and its structure was identified by MS, ^1^H NMR, and ^13^C NMR. SYS18 with a purity greater than 95% was used for subsequent biological experiments. NMR data, mass spectrometry data, and purity information of SYS18 can be found in Figures S1–S3.

### 3.2 SYS18 Inhibits Acute Peripheral Inflammation Induced by Acetic Acid in Mice

Acetic acid‐induced increased peritoneal capillary permeability in mice is a classic model of peripheral inflammation. Anti‐inflammatory effects of SYS18 were evaluated by using an increased peritoneal capillary permeability assay. After intravenous injection of Evans blue saline solution, intraperitoneal injection of acetic acid was used to increase peritoneal capillary permeability in mice. The peritoneal capillary permeability of mice in each group was compared by collecting peritoneal lavage fluid and measuring abdominal Evans blue absorbance.

**FIGURE 3:**
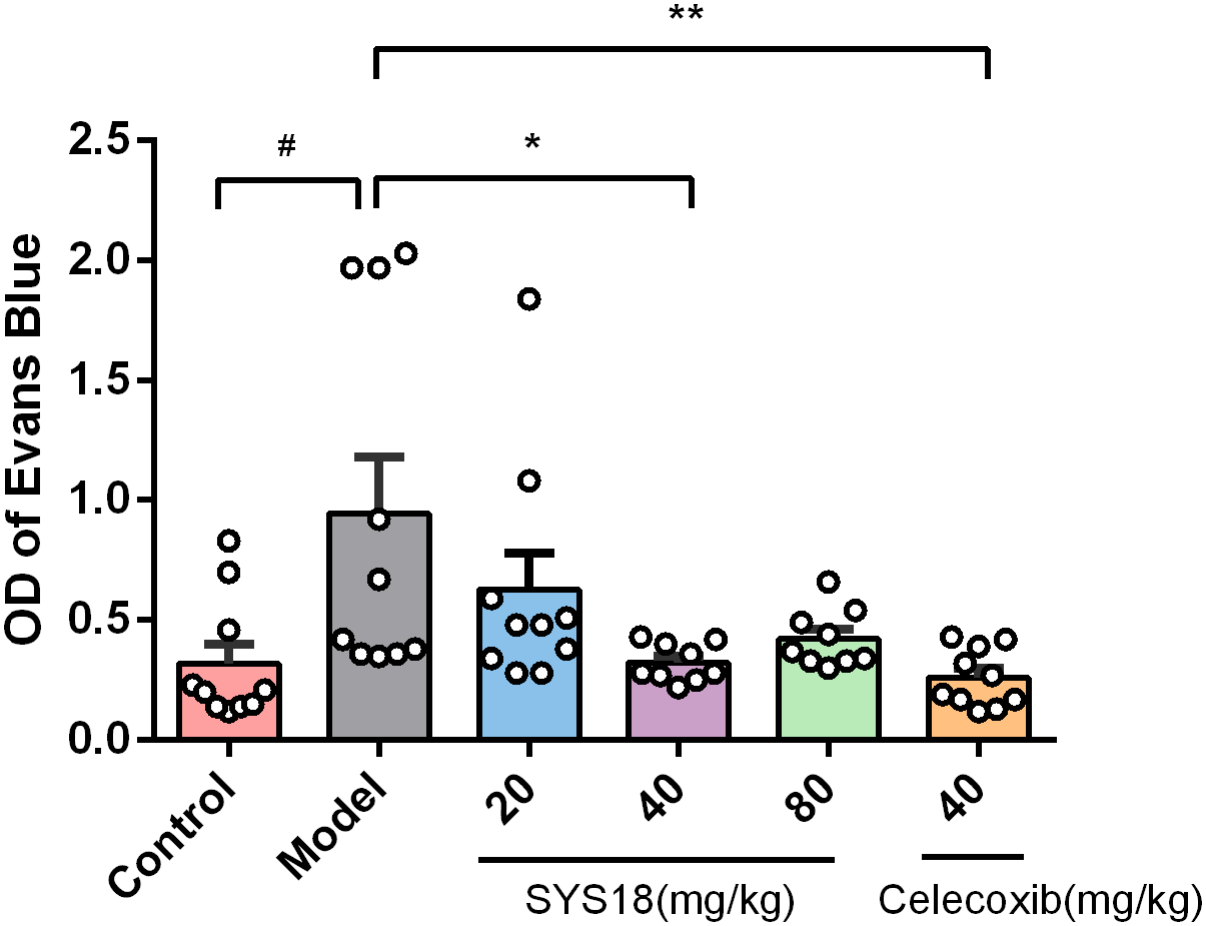
Anti‐inflammatory effects of SYS18 on acetic acid‐induced peritoneal capillary permeability increase models; *n* = 9–10; data are presented as mean ± SD. Data were analyzed by Mann–Whitney *U* test or Kruskal–Wallis rank sum test, ^#^ *p* < 0.05 vs. control group; *p < 0.05 vs. model group; **p < 0.01 vs. model group.

As shown in the Figure 3 (Kruskal–Wallis rank sum test, *p* = 0.0024), the absorbance of Evans blue in the model group was significantly increased (*p* < 0.05), indicating that the peritoneal capillary permeability of mice was significantly increased. Compared with the model group, low‐dose SYS18 did not significantly reduce the absorbance of Evans blue. On the contrary, SYS18 at medium dose significantly reduced the concentration of Evans blue in the peritoneal lavage fluid of mice, indicating that SYS18 at medium dose significantly inhibited the increase in peritoneal capillary permeability induced by acetic acid in mice. Although there was no statistical difference between the high‐dose group and the model group, a significant reduction trend could be observed. These results suggest that SYS18 has a certain therapeutic effect on acute inflammation.

When cerebral ischemic stroke occurs, oxidative stress and inflammatory factors can destruct BBB integrity and result the serious brain damage [24].

### 3.3 SYS18 Has Neuroprotective Effects in MACO Rats

The neuroprotective effect of SYS18 was verified by experiments of neurological scores in rats, brain edema in rats and mice, and histological damage of nerve cells in rats. The results showed that SYS18 exhibited a good neuroprotective effect.

#### 3.3.1 SYS18 Alleviates Neurological Scores in MCAO Rats

**FIGURE 4:**
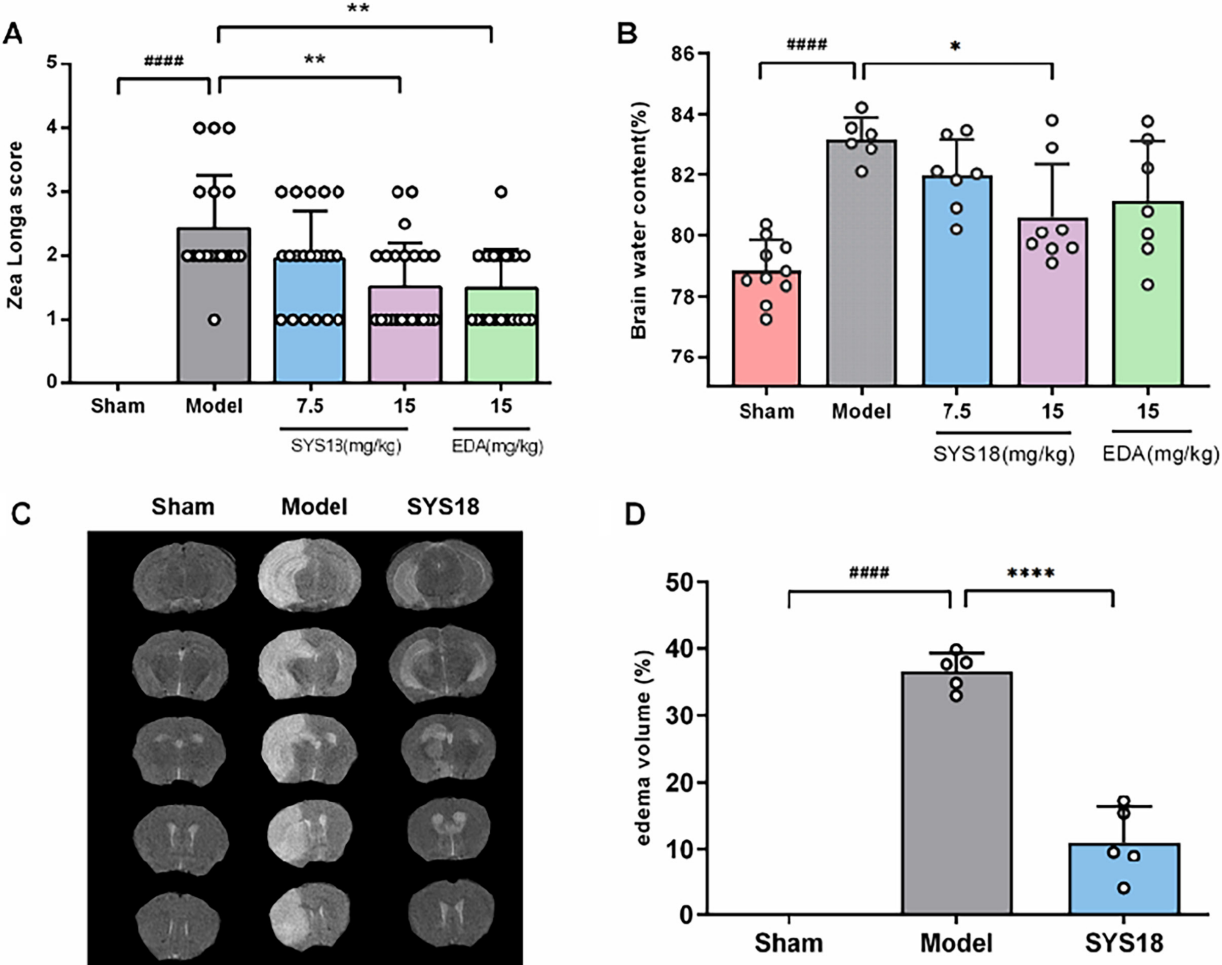
SYS18 alleviated neurological injury and cerebral edema. (A) The neurological status of the animals was evaluated by Zea‐Longa scoring criteria. *n* = 19–24, data were analyzed by Mann–Whitney *U* test or Kruskal–Wallis rank sum test. (B) SYS18 reduced brain water content after ischemic stroke, *n* = 6–10. (C) Brain edema volume after ischemic stroke in T2‐weighted MRI, *n* = 3, SYS18 = 30 mg/kg. (D) Brain edema volume percentage in T2‐weighted MRI. Results were expressed as mean ± SD. Measurement data were analyzed by one‐way analysis of variance combined with Dunnett's multiple comparison method. ^####^ *p* < 0.0001 vs. sham group; *p < 0.05 vs. model group; **p < 0.01 vs. model group; ****p < 0.0001 vs. model group.

The therapeutical effects of SYS18 on neurological dysfunction were evaluated by the neurological scoring experiment. As shown in Figure 4A (Kruskal–Wallis rank sum test, *p* = 0.0008), the neurological score of model group (2.42 ± 0.84) significantly increased compared with the sham group (0.00 ± 0.00) (*p* < 0.0001). SYS18 in low‐dose group (1.95 ± 0.74) reduced neurological scores insignificantly (*p* > 0.05). On the contrary, SYS18 in high‐dose group (1.52 ± 0.68) and edaravone (1.50 ± 0.61) could significantly reduce neurological scores compared to the model group (*p* < 0.01). It indicated that SYS18 can ameliorate neurological lesion induced by cerebral ischemic injury.

#### 3.3.2 SYS18 Alleviates Cerebral Edema in MCAO Mice and Rats

It has been documented that space‐occupying cerebral edema usually appears the day after the onset of stroke and may last for 48 h or even longer [25]. If effective measures are not taken, it may lead to irreversible neuronal damage or even brain death [26]. However, there is still no effective drug for the treatment of brain edema, and the development and research of drugs for treating brain edema are imminent [27]. Here, we evaluated the therapeutic effects of SYS18 on ischemic brain edema in rats and mice in different ways.

Twenty four to forty eight hours is a rapidly progressive course of cerebral edema [28]. The brain water content is the important indicator to evaluate the severity of cerebral edema. Therefore, we had tested the brain dry‐wet weight ratio to investigate the effects of SYS18 on cerebral edema in MCAO rats. As shown in Figure 4B (one‐way ANOVA, *F*_(3, 24)_ = 3.631, *p* = 0.0272), compared with the sham group (78.85% ± 0.97%), the brain water content increased in model group (83.18% ± 0.71%) (*p <* 0.0001). The SYS18 in low‐dose group (81.98% ± 1.18%) decreased the brain water content insignificantly (*p* > 0.05). On the contrary, SYS18 in high‐dose group (80.62% ± 1.73%) could significantly reduce the water content compared to the model group (*p <* 0.05). It is better than edaravone (81.13% ± 1.98%).

Quantification of mice brain edema by T2‐MRI showed the same results. After occlusion of cerebral‐blood flow, the brain edema volume increased to 36.64% ± 2.69%. However, the brain edema volume remarkably decreased to 11.01% ± 5.32% after administration of high‐dose SYS18 (*p <* 0.0001) (Figure 4C,D). This experiment showed that SYS18 had effect for the treatment of cerebral edema.

#### 3.3.3 SYS18 Improves Serum Biochemical Indicators in MCAO Cerebral Edema Models

**FIGURE 5:**
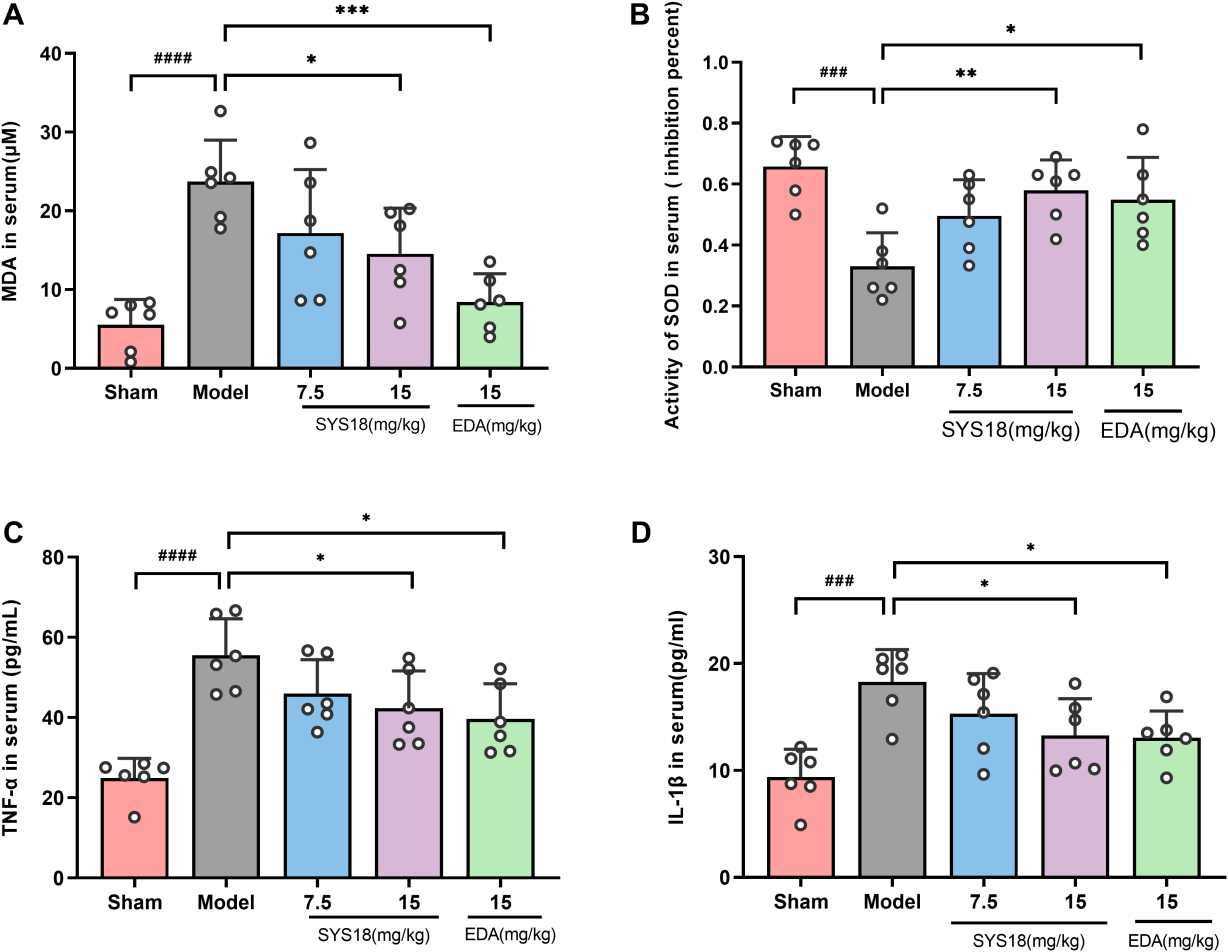
Effects of SYS18 on serum biochemical indicators in MCAO cerebral edema model. (A) MDA content in serum; (B) SOD activity in serum; (C) TNF‐α content in serum; (D) IL‐1β content in serum; *n* = 6. Data were presented as mean ± SD. Measurement data were analyzed by one‐way analysis of variance combined with Dunnett's multiple comparison method, ^###^ *p* < 0.001 vs. sham group; ^####^ *p* < 0.0001 vs. sham group; *p < 0.05 vs. model group; **p < 0.01 vs. model group; ***p < 0.001 vs. model group.

In order to further study the effects of SYS18 on oxidative stress and inflammation in MCAO rats, the levels of MDA and SOD were detected by kit and the levels of TNF‐α and IL‐1β in serum were detected by ELISA. As shown in Figure 5A,B (one‐way ANOVA, *F*_(3, 20)_ = 6.930, *p* = 0.0022; one‐way ANOVA, *F*_(3, 20)_ = 5.345, *p* = 0.0072), the results showed that the levels of MDA in serum of MCAO group significantly increased compared with the sham group (*p* < 0.0001). Treatment with high‐dose group SYS18 reduced MDA levels in serum significantly (*p* < 0.05). On the other hand, the levels of SOD in serum significantly decreased in the MCAO group (*p* < 0.001). Treatment with high‐dose SYS18 and edaravone could improve SOD level in serum.

Inflammation accelerates the damage of BBB in cerebral edema. TNF‐α and IL‐1β are important indicator of inflammation. As shown in Figure 5C,D (one‐way ANOVA, *F*_(3, 20)_ = 3.660, *p* = 0.0298; one‐way ANOVA, *F*_(3, 20)_ = 3.434, *p* = 0.0366), TNF‐α and IL‐1β significantly increased in model group compared the sham group (*p* < 0.0001 or 0.001). However, TNF‐α and IL‐1β significantly decreased after administration of high‐dose SYS18 group and edaravone (*p* < 0.05). These results suggested that SYS18 could inhibit oxidative stress and inflammation, which might be beneficial to the therapeutical effect against AIS. And we hypothesized that the therapeutic effect of SYS18 on acute inflammation may be related to the decreased expression of related inflammatory factors.

#### 3.3.4 SYS18 Attenuates the Histological Damage of Neural Cells in MCAO Rats

**FIGURE 6:**
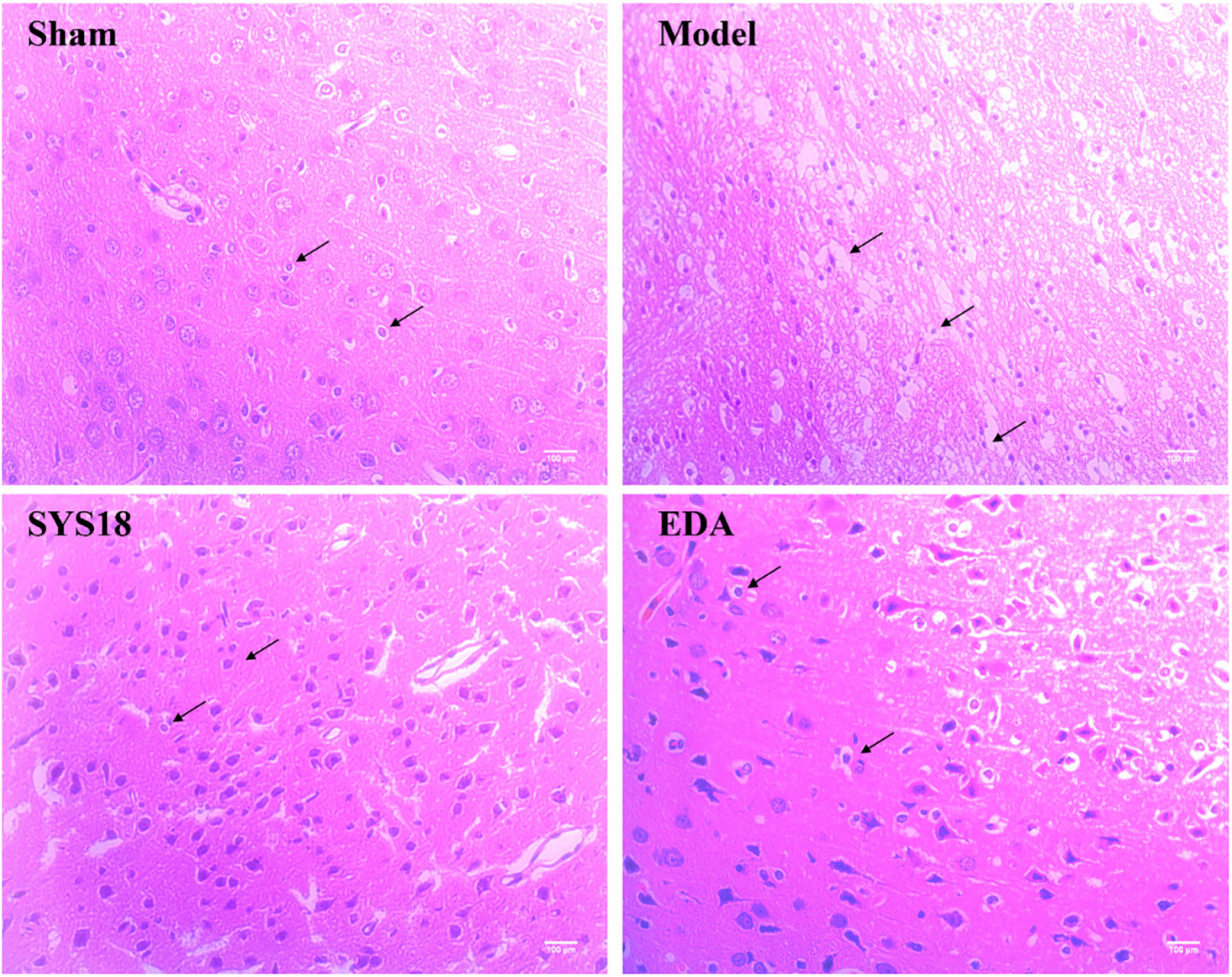
SYS18 improved neural tissue morphology in the cerebral cortex after ischemia–reperfusion. The cytoplasm was stained in red, and the nucleus was stained in blue. *n* = 3, observe in 400× inverted microscope, scale bar = 100 μm.

Neurological function deterioration appeared after acute ischemic stroke, which may lead to irreversible damage to cranial nerves or even neuron death if effective interventions are not taken [26]. Hematoxylin–eosin staining was used to observe the pathological changes in neural tissue after MCAO. High‐dose SYS18 showed preferably anti‐cerebral edema effect; thus, we took high‐dose SYS18 for further study. In the sham group, the morphology of nerve cells in cortex was oval and arranged tightly. After MCAO surgery, the amount of nerve cells decreased, and the cells were disorganized and the nuclei was pyknotic. Besides, the intercellular space increased and more vacuoles were formed. After administration of SYS18, the necrosis of nerve cell in the brain tissue was attenuated. Besides, a number of cells were able to retain normal morphology, and the amount of pyknotic nuclei were reduced (Figure 6). These results indicated that SYS18 could alleviate the morphological damage of nerve cells induced by cerebral ischemia–reperfusion.

### 3.4 SYS18 Protects the Integrity of BBB in MACO Rats

Oxidative stress and inflammatory factors can produce various harmful components which destruct the integrity of BBB [24]. The main feature of this process is the increasing of BBB permeability. Recent study showed that the change in BBB permeability is correlated with the degradation of endothelial glycocalyx. Inflammatory factors play important roles in both glycocalyx degradation and BBB permeability change [29]. In the present study, we demonstrated that SYS18 protects BBB integrity by reduces permeability and glycocalyx degradation.

#### 3.4.1 SYS18 Decreases BBB Permeability in MCAO Rats

**FIGURE 7:**
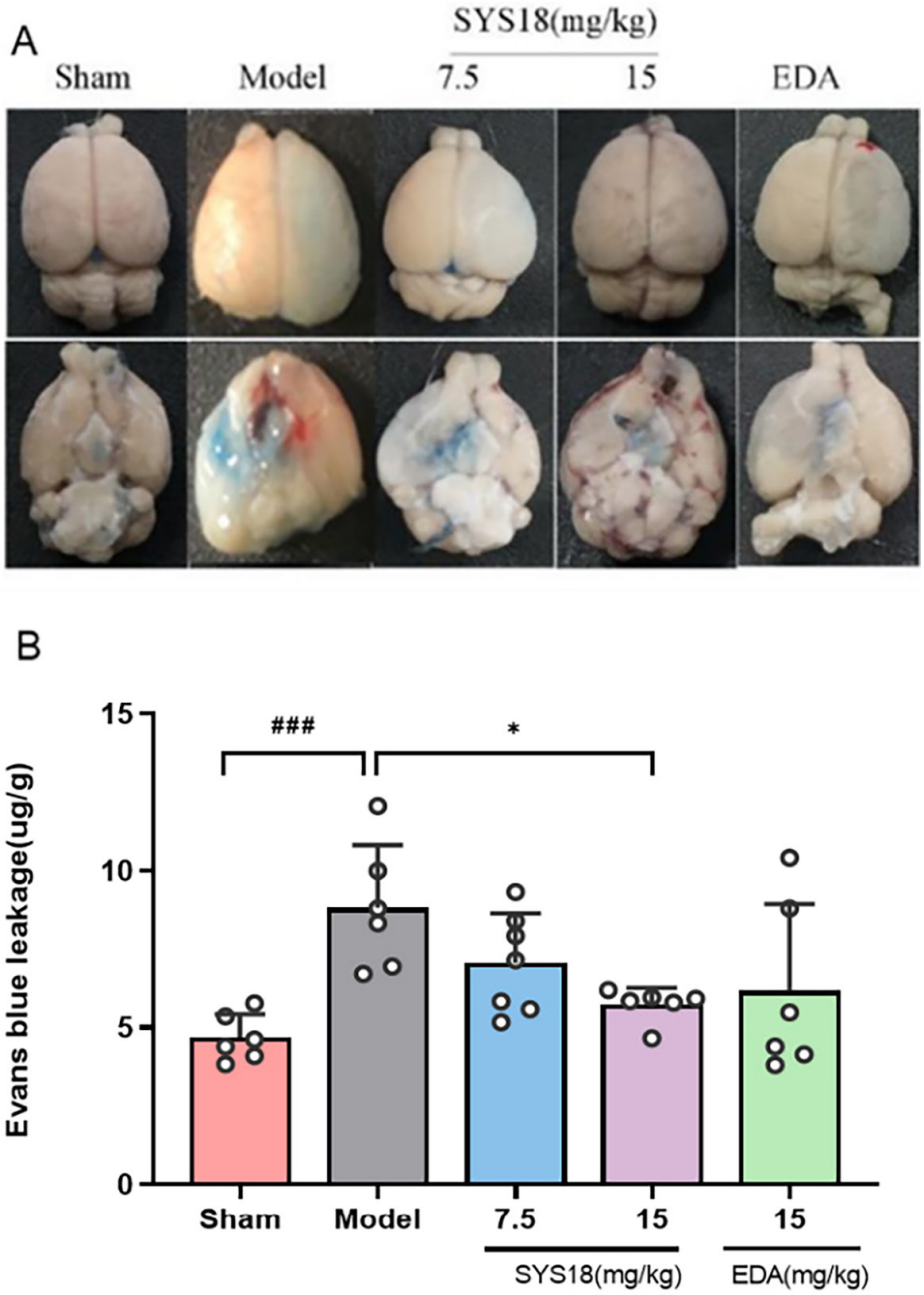
SYS18 attenuated BBB damage after ischemia–reperfusion injury. (A) Representative image of Evans blue content in brain. (B) Evans blue content in brain. BBB permeability was evaluated by Evans blue extravasation. *n* = 6–7. Data were expressed as mean ± SD. Measurement data were analyzed by one‐way analysis of variance combined with Dunnett's multiple comparison method. ^###^ *p* < 0.001 vs. sham group; *p < 0.05 vs. model group.

The BBB permeability is an important indication to reflect whether the blood–brain barrier is damaged. In order to evaluate the protection of SYS18 for BBB permeability in acute ischemic stroke, we measured the Evans blue content in the brain tissue. In the model group, Evans blue content significantly increased compared with the sham group (*p* < 0.001). Comparing with the MCAO model group, SYS18 in low‐dose group (7.06 ± 1.58 μg/g) decreased Evans blue content in brain tissue insignificantly (*p* > 0.05). On the contrary, treatment with high‐dose SYS18 (5.74 ± 0.54 μg/g) significantly inhibited penetration of Evans blue into brain (*p* < 0.05). Comparing with the positive control (6.20 ± 2.76 μg/g), the effect of high‐dose SYS18 was better (Figure 7A,B, one‐way ANOVA, F _(3, 21)_ = 3.121, *p* = 0.0478). These results indicated that SYS18 might alleviates the disruption of BBB.

#### 3.4.2 SYS18 Mitigates the Degradation of Endothelial Glycocalyx in MCAO Rats

**FIGURE 8:**
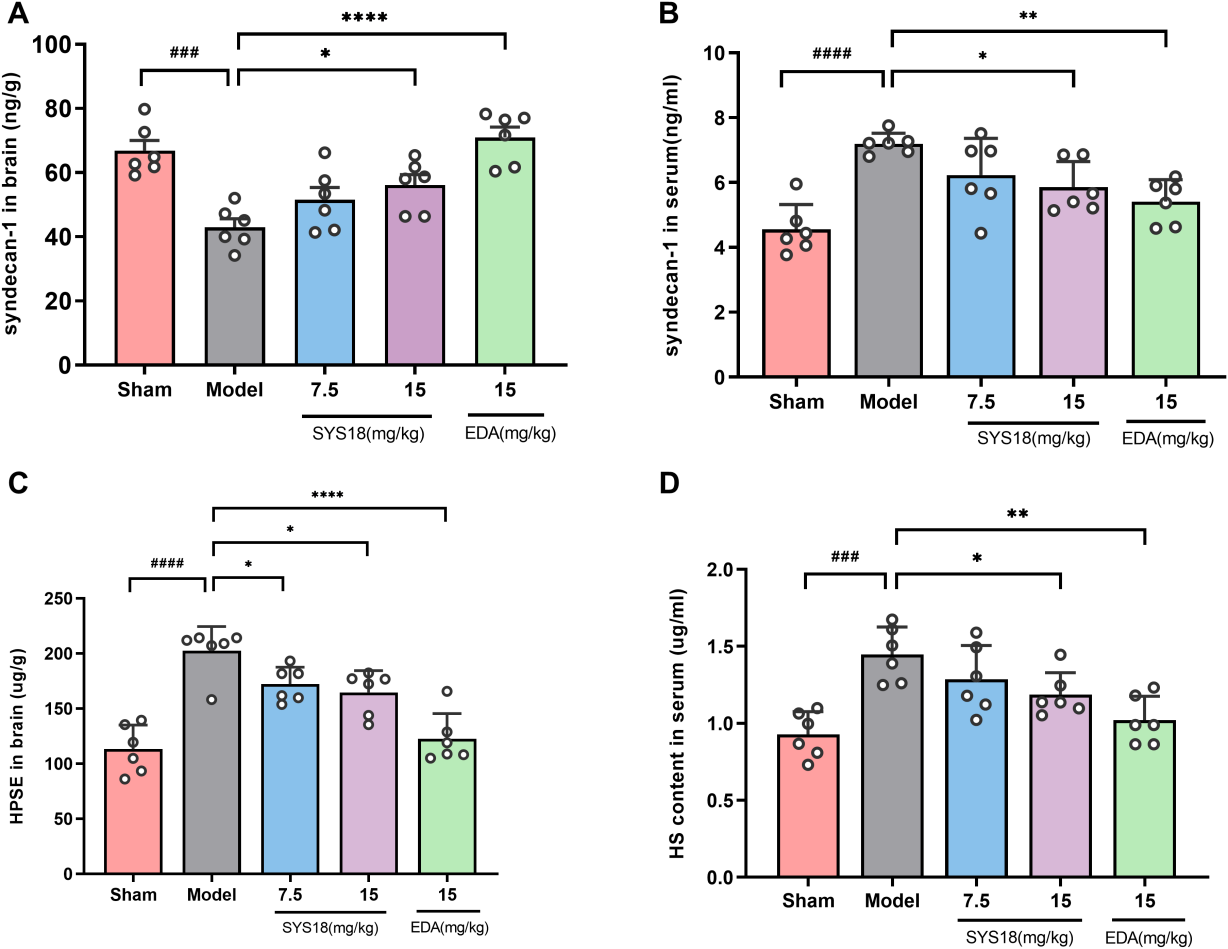
Effects of SYS18 on endothelial glycocalyx in ischemia–reperfusion injury. (A) syndecan‐1 content in brain; (B) syndecan‐1 content in serum; (C) HPSE content in brain; (D) HS content in serum; *n* = 6. Data were presented as mean ± SD and were analyzed by one‐way analysis of variance combined with Dunnett's multiple comparison method. ^###^ *p* < 0.001 vs. sham group; ^####^ *p* < 0.0001 vs. sham group; *p < 0.05 vs. model control group; **p < 0.01 vs. model control group, ****p < 0.0001 vs. model control group.

The glycocalyx is a glycoprotein on the surface of the vascular endothelium, and the glycocalyx core protein is bound to the cell membrane by syndecans, and heparin sulfate (HS) is a major part of the negatively charged glycosaminoglycan side chain. When cerebral ischemia occurs, endothelial glycocalyx degradation leads to endothelial dysfunction and BBB damage [30, 31]. It has been shown that syndecans decreased and heparanase (HPSE) increased in ischemic brain tissue [9]. In order to research the effect of SYS18 on endothelial glycocalyx in cerebral ischemia, syndecan‐1, HS, and HPSE were examined in brain tissue or serum. After 48 h of ischemia–reperfusion, comparing with the sham group, the syndecan‐1 content of the model group was significantly decreased in brain (*p* < 0.001), and the HPSE content in brain of the model group was significantly increased along with HS increased in serum (*p* < 0.0001 or 0.001). The change in syndecan‐1 and HS in SYS18 low‐dose group was not insignificantly (*p* > 0.05). Comparing with the MCAO model group, SYS18 high‐dose group significantly increased syndecan‐1 in brain tissue and decreased HPSE in brain tissue and HS in serum (*p* < 0.05) (Figure 8A–D, one‐way ANOVA, *F*_(3, 20)_ = 12.71, *p* < 0.0001; one‐way ANOVA, *F*_(3, 20)_ = 5.596, *p* = 0.0059; one‐way ANOVA, *F*_(3, 20)_ = 15.90, *p* < 0.001; one‐way ANOVA, *F*_(3, 20)_ = 6.162, *p* = 0.0039).

The above results showed that after ischemia–reperfusion, glycocalyx components were degraded, as shown by the decrease in syndecan‐1 content in brain tissue and the entry of shed syndecan‐1 into the blood circulation, resulting in the increase of blood syndecan‐1. After ischemia–reperfusion, the expression of HPSE in brain tissue increases, and HPSE can specifically degrade HS. Degradation of HS causes HS to fall off into blood circulation and increase the content of HS in serum. However, after SYS18 intervention, it was able to increase sydecan‐1 in brain tissue, reduce HS shedding, which indicated that SYS18 can reduce the degradation of endothelial glycocalyx in brain tissue, and it may play a protective role on the blood–brain barrier.

#### 3.4.3 SYS18 Regulates Tight Junction Proteins Claudin‐5 and MMP‐9 Expression in MCAO Rats

Disruption of BBB integrity and degradation of tight junction proteins (TJs) are implicated, which is related to downregulation of TJs and upregulation of MMP‐9 [32, 33].

**FIGURE 9:**
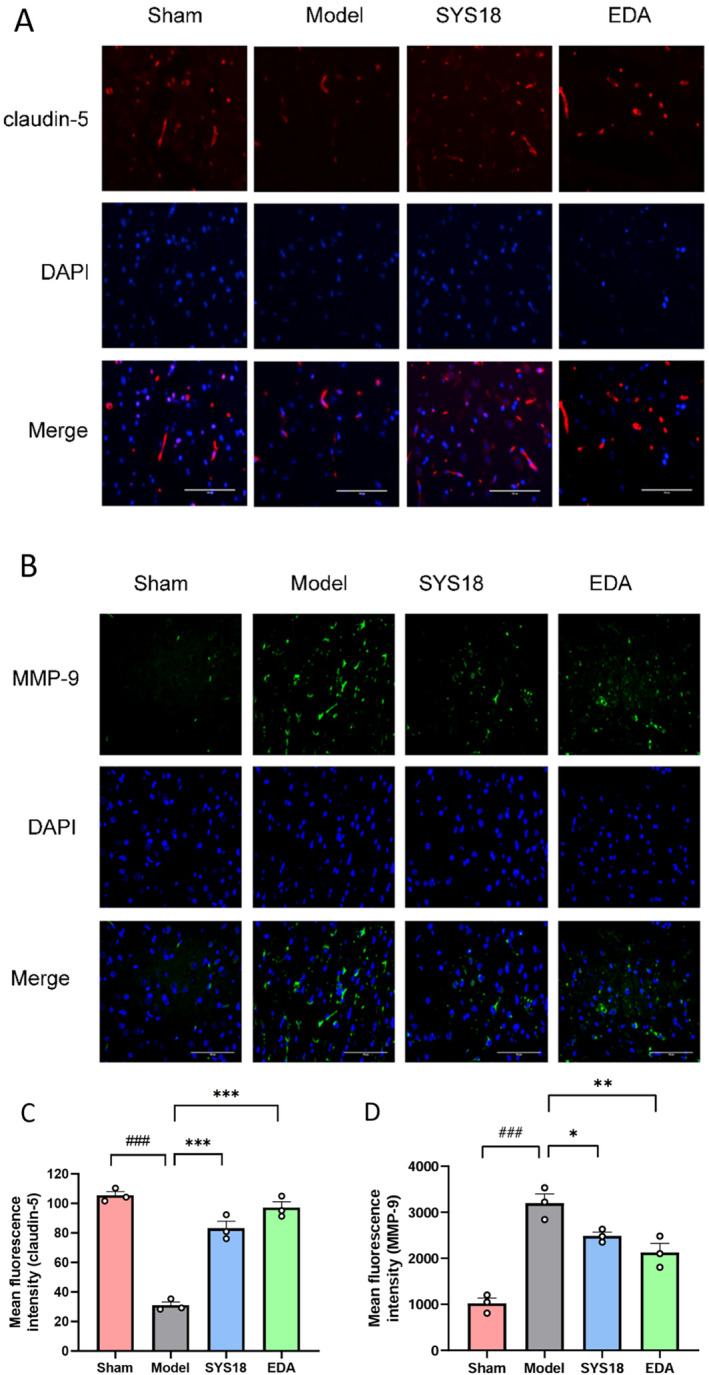
(A) Immunofluorescence of claudin‐5 expression in each model; (B) Immunofluorescence of MMP‐9 expression in each model. Scale bar = 100 μm (observe in 400× inverted microscope); (C) Mean fluorescence intensity of claudin‐5; (D) Mean fluorescence intensity of MMP‐9. ^###^ *p* < 0.001 vs. sham group; *p < 0.05 vs. model group; **p < 0.01 vs. model group; ***p < 0.001 vs. model group.

Claudin‐5 is an important part of TJs. It shows high levels in vascular endothelial cells and, as the main cell adhesion molecule of TJs, plays an important role in the tightness of the BBB [34]. We further investigated whether the levels of TJs could be regulated by SYS18. As shown in the immunofluorescence experiments, the fluorescent intensity of claudin‐5 decreased in the model group compared with sham group, while fluorescent intensity increased after treatment with high‐dose SYS18 (Figure 9A,C, one‐way ANOVA, F _(2, 6)_ = 81.75, *p* < 0.0001).

Substantial evidence confirms that MMP9 expression degrades the basement membrane and extracellular matrix in MCAO rats [33]. Therefore, we finally examined whether SYS18 affecting BBB structures is associated with MMP9. The fluorescent intensity of MMP‐9 increased in the model group comparing with sham, while it decreased after treatment with high‐dose SYS18 (Figure 9B,D, one‐way ANOVA, *F*_(2, 6)_ = 10.47, *p* = 0.0110). These results suggested that SYS18 could attenuate degradation of claudin‐5 and inhibit the expression of MMP‐9. Therefore, SYS18 played an important role in sustaining BBB integrity.

### 3.5 Pharmacokinetic Study

**FIGURE 10:**
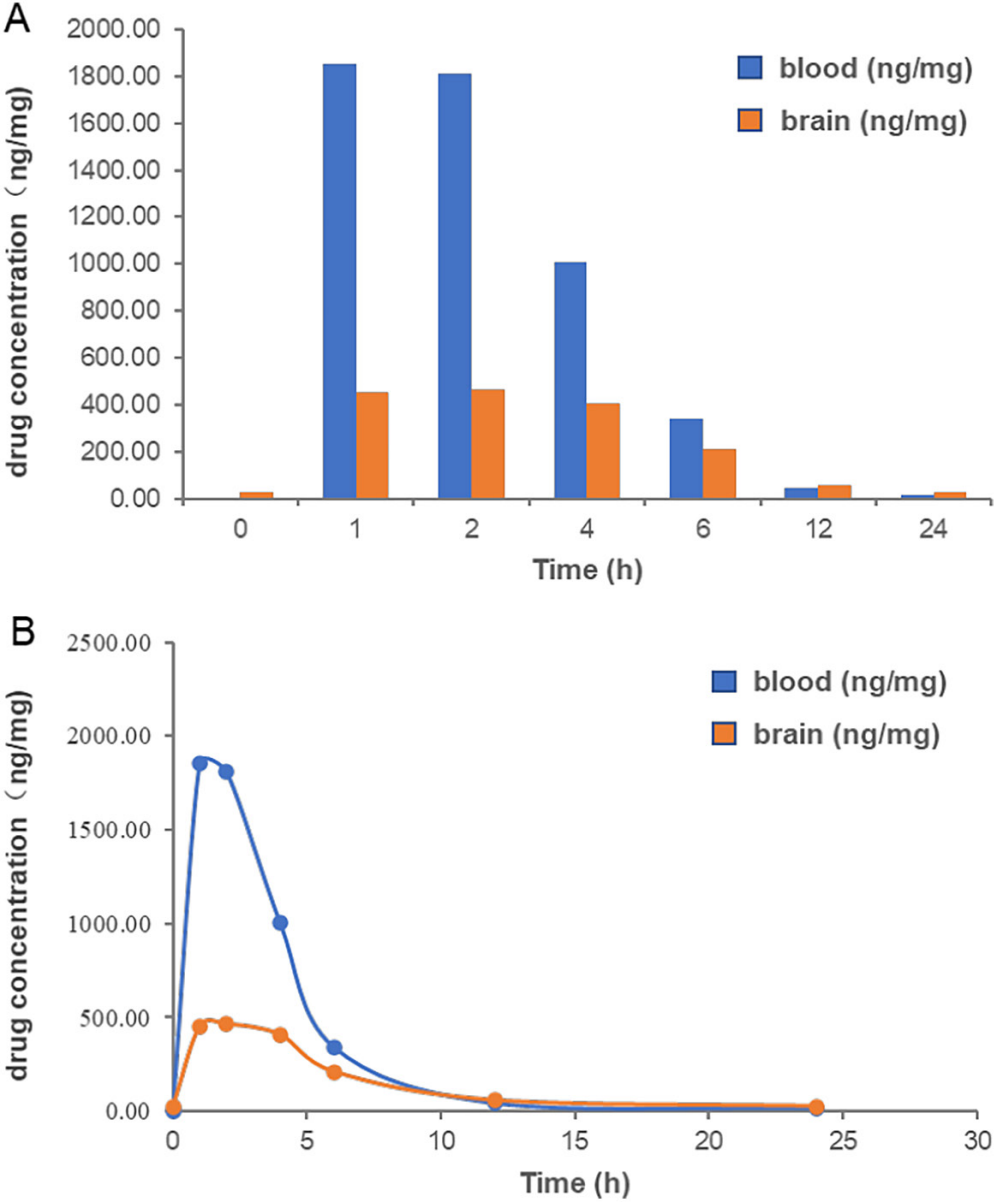
Blood and brain drug concentrations in rats after intraperitoneal injection of SYS18 (A) Comparison of SYS18 concentrations in blood and brain; (B) drug‐time curve of the SYS18 in the blood and brain.

In former study, we have measured the blood pharmacokinetics of SYS18 in oral and intravenous rats, which showed good drug‐like properties. To further explore the potential of SYS18 as a central nervous system drug, pharmacokinetic (PK) experiments were conducted in SD rats in blood and brain. As shown in Figure 10A,B and Table 1, after intraperitoneal injection of SYS18, the area under the curve (AUC_0‐t_) was 8444.69 and 3504.387 ng/mg, respectively, suggesting that the administration was appropriate. The cerebral‐blood ratio of area under the curve (AUC_0‐t_) was greater than 0.4, and the B/P was greater than 0.2 at 1–2 h and greater than 0.4 at 4–6 h, indicating that SYSY18 has good BBB permeability and has the potential as a central nervous system drug. The peak time of SYS18 in blood and brain was 1 and 2 h, respectively. The half‐life were 3.174 and 5.125 h, respectively, indicating good metabolic stability.

**TABLE 1:** PK parameters in blood and brain of rats after intraperitoneal injection of SYS18.

Parameter	Unit	Value (blood)	Value (brain)
Lambda_z	1/h	0.218	0.135
*t*_1/2_	h	3.174	5.125
*T*_max_	h	1	2
*C*_max_	ng/mg	1851.014	463.687
Tlag	h	0	0
Clast_obs/*C*_max_	/	0.009	0.057
AUC_0‐t_	ng/mg × h	8444.690	3504.387
AUC_0‐inf_obs_	ng/mg × h	8522.639	3700.110
AUC_0‐t/0‐inf_obs_	/	0.991	0.947
AUMC_0‐inf_obs_	ng/mg × h^2^	32940.745	26458.752
MRT_0‐inf_obs_	h	3.865	7.151
Vz/F_obs	(mg/kg)/(ng/mg)	0.003	0.010
Cl/F_obs	(mg/kg)/(ng/mg)/h	0.001	0.001

## 4 Conclusion

In the early stage of acute ischemic stroke (AIS), cerebral ischemia and hypoxia lead to oxidative stress which results breakdown of the blood–brain barrier and neuroinflammation. Neuroinflammation exacerbate cerebral edema. Anti‐oxidative stress and anti‐inflammatory treatment can protect the BBB to reduce cerebral edema. Given that SYS18 is known to have excellent antioxidant activity and bioavailability and lower toxicity, we aimed to enhance its associated BBB protection and anti‐inflammatory activity research.

Therefore, we constructed a more severe model of middle cerebral artery occlusion (MCAO)‐induced cerebral edema, comprehensively and systematically elucidated anti‐edema and anti‐inflammatory effects of SYS18. The results confirmed its neuroprotective effects against cerebral edema by reducing neurological function scores, alleviating cerebral edema, improving biochemical indicators (reducing the expression of inflammatory factors), and mitigating cerebral tissue pathological damage. Simultaneously, by reducing BBB permeability in cerebral tissue, inhibiting glycocalyx degradation, and regulating the expression of BBB tight junction proteins matrix metalloproteinase‐9 (MMP‐9) and claudin‐5, we demonstrated its protective effects on BBB integrity. We evaluated its broad‐spectrum anti‐inflammatory effects in a mouse model with increased peritoneal capillary permeability. Pharmacokinetic experiments related to this study first demonstrated that SYS18 has excellent BBB permeability.

The high bioavailability and good BBB permeability of SYS18 ensure that the compound can also exert normal physiological effects in the brain. The inhibitory effect of SYS18 on neuritis may be related to the protection of BBB integrity and the reduction of TNF‐α and IL‐1β in the brain. The protection of BBB integrity is closely related to the inhibition of glycocalyx degradation by SYS18. Through these neuroprotective effects, SYS18 can alleviate brain edema injury and neurological dysfunction in AIS. Based on these findings, SYS18 has immense potential to become a neuroprotective agent in the future.

## Conflicts of Interest

The authors declare no conflicts of interest.

## Supporting information


Figure S1.

## Data Availability

The data that support the findings of this study are available from the corresponding author upon reasonable request.
