# Supplementary material for: The Neuroprotection of 1,2,4‐Triazole Derivative by Inhibiting Inflammation and Protecting BBB Integrity in Acute Ischemic Stroke
Source: CNS Neurosci Ther. 2024 Nov 5;30(11):e70113. doi: 10.1111/cns.70113 (PMC11537802; doi:10.1111/cns.70113)
Supplement: Supplementary file 1 — Figure S1. [file CNS-30-e70113-s001.docx]

Supporting Information

**The neuroprotection of 1,2,4-Triazole derivative by inhibiting inflammation and protecting BBB integrity in acute ischemic stroke**

Xuan Liu^1^, Jingning Luo^1^, Jianwen Chen^1^, Ping Huang, Gongyun He, Xueshi Ye, Ruiqi Su, Yaoqiang Lao, Yang Wang, Xiangjun He, Jingxia Zhang*

Department of Medicinal Chemistry, School of Pharmaceutical Science, Sun Yat-sen University, Guangzhou, 510006, PR China

* Corresponding author. Mail address: zhjingx@mail.sysu.edu.cn

^1^These three authors contributed equally to this paper.

**Table of Contents**

**1. NMR spectra of SYS18 S2**

**2. HRMS result of SYS18 S3**

**3. HPLC result of SYS18 S4**

**1. NMR spectra of SYS18**


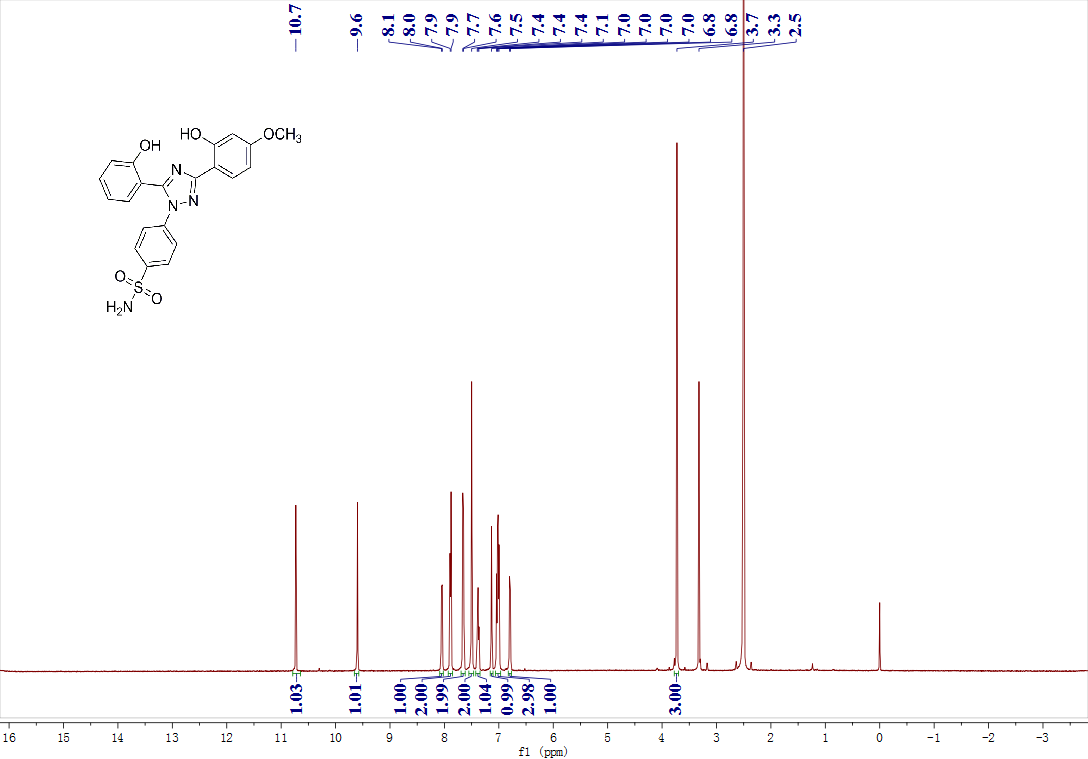


Figure 1S. ^1^H NMR (500 MHz, DMSO-*d_6_*) of SYS18.


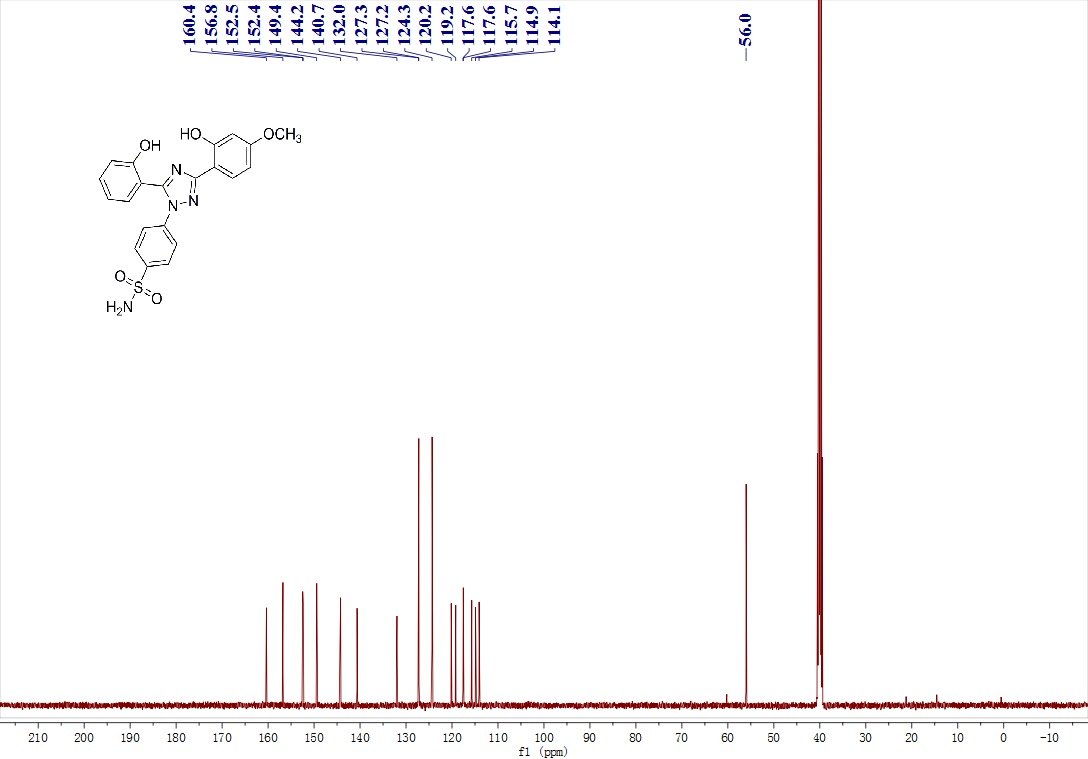


Figure 2S. ^13^C NMR (126 MHz, DMSO-*d_6_*) of SYS18.

**2. HRMS result of SYS18**


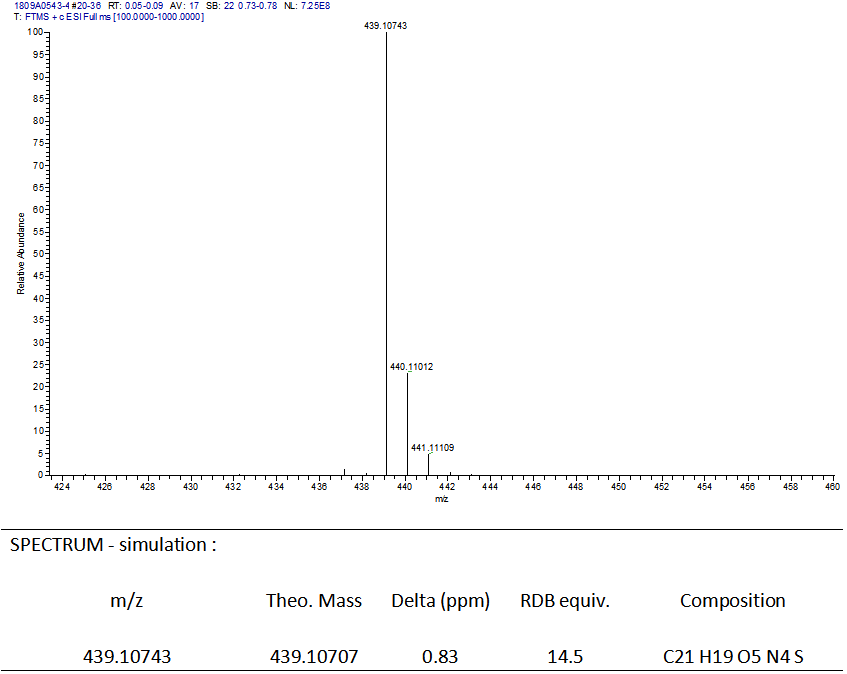


Figure 3S. HRMS of SYSY18.

**3. HPLC result of SYS18**


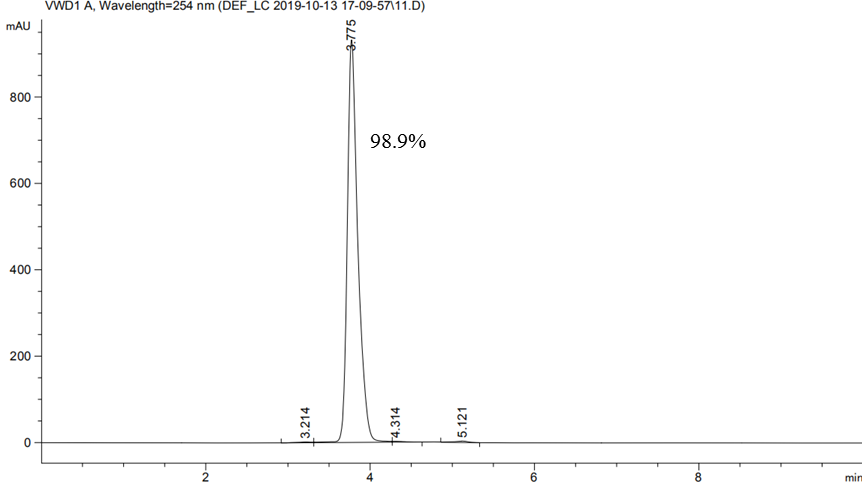


Figure 4S. HPLC of SYS18**.**
